# Age‐specific incidence, risk factors and outcome of acute abdominal aortic aneurysms in a defined population

**DOI:** 10.1002/bjs.9838

**Published:** 2015-05-07

**Authors:** D. P. J. Howard, A. Banerjee, J. F. Fairhead, A. Handa, L. E. Silver, P. M. Rothwell, the Oxford Vascular Study

**Affiliations:** ^1^ Stroke Prevention Research Unit, Nuffield Department of Clinical NeurosciencesUniversity of OxfordOxfordUK; ^2^ Department of Vascular SurgeryOxford University Hospitals NHS TrustOxfordUK; ^3^ Centre for Cardiovascular SciencesUniversity of BirminghamBirminghamUK

## Abstract

**Background:**

Contemporary population‐based data on age‐specific incidence and outcome from acute abdominal aortic aneurysm (AAA) events are needed to understand the impact of risk factor modification and demographic change, and to inform AAA screening policy.

**Methods:**

In a prospective population‐based study (Oxfordshire, UK, 2002–2014), event rates, incidence, early case fatality and long‐term outcome from all acute AAA events were determined, both overall and in relation to the four main risk factors: smoking, hypertension, male sex and age.

**Results:**

Over the 12‐year interval, 103 incident acute AAA events occurred in the study population of 92 728 (men 72·8 per cent; 59·2 per cent 30‐day case fatality rate). The incidence per 100 000 population per year was 55 in men aged 65–74 years, but increased to 112 at age 75–84 years and to 298 at age 85 years or above. Some 66·0 per cent of all events occurred in those aged 75 years or more. The incidence at 65–74 years was highest in male smokers (274 per 100 000 population per year); 27 (96 per cent) of 28 events in men aged less than 75 years occurred in ever‐smokers. Mean(s.d.) age at event was lowest in current smokers (72·2(7·2) years), compared with that in ex‐smokers (81·2(7·0) years) and never‐smokers (83·3(7·9) years) (P < 0·001). Hypertension was the predominant risk factor in women (diagnosed in 93 per cent), with 20 (71 per cent) of all 28 events in women occurring in those aged 75 years or above with hypertension. The 30‐day case fatality rate increased from 40 per cent at age below 75 years to 69 per cent at age 75 years or more (P = 0·008).

**Conclusion:**

Two‐thirds of acute AAA events occurred at age 75 years or above, and more than 25 per cent of events were in women. Taken with the strong associations with smoking and hypertension, these findings could have implications for AAA screening.

## Abstract 2

Comprehensive cohort study

## Introduction

Abdominal aortic aneurysm (AAA) causes approximately 4000 deaths per year in England and Wales^1^, and more than 175 000 deaths globally. Ruptured AAA accounts for 1 per cent of deaths in men aged over 65 years^2^. AAAs usually remain undetected until rupture. Rupture carries an 80 per cent mortality rate^3^, ^4^, ^5^, compared with a 30‐day mortality rate of only 2–6 per cent for elective surgical repair of non‐ruptured AAAs^6^, ^7^. Population screening to detect and repair AAAs before rupture may reduce AAA‐related mortality, as shown in the UK Multicentre Aneurysm Screening Study (MASS)^4^, ^8^, ^9^. National screening programmes have recently been initiated for men aged 65 years in England^10^, Wales, Northern Ireland, Scotland^11^, Sweden^12^ and the USA^13^.

However, the prevalence of AAA in men aged 65–74 years is in steep decline^10^, ^14^, ^15^, and AAA rupture may be shifting to older age groups^14^, ^15^, ^16^, ^17^, ^18^. This raises concerns that one‐off screening of men at age 65 years may in future have limited impact on overall AAA‐related mortality, particularly in light of increasing life expectancy^9^, ^19^, ^20^.

A greater understanding of the epidemiology, risk factors and outcome of acute AAA events at a population level will inform future screening programme strategy. Smoking, hypertension, male sex and age are the key risk factors for AAA formation^21^, ^22^, ^23^, ^24^, ^25^, ^26^. Smoking and hypertension may additionally increase the risk of rupture^27^, ^28^, ^29^, but contemporary population‐based incidence rates for acute AAA events in these risk groups have not been determined. Previous studies have been based on routinely collected hospital coding data^3^, ^14^, ^15^, ^16^, ^17^, ^18^, ^28^, ^30^, ^31^, ^32^ and retrospective registries^33^, ^34^, or restricted to prevalent groups, limited by age and sex, or cohorts of volunteers with specific risk factor profiles^4^, ^19^, ^22^, ^26^, ^35^, ^36^, ^37^, ^38^, ^39^, ^40^. Rates of acute AAA events are also unknown for women with hypertension or smoking history, who are potentially at high risk of aneurysm formation.

The aim of the present study was to determine event rates, incidence, early case fatality and long‐term outcome of all acute AAA events during 2002–2014 (before the introduction of national screening in the region), in a prospective population‐based study in Oxfordshire, UK.

## Methods

The Oxford Vascular (OXVASC) Study was approved by the local research ethics committee. The OXVASC Study population comprises all individuals (12‐year average 92 728), irrespective of age, registered with approximately 100 family physicians in nine general practices in Oxfordshire, UK^41^. In the UK, the majority of individuals register with a general practice, which provides their primary healthcare and holds a lifelong record of all medical consultations, and details of medications, BP measurements and investigations. All participating practices held accurate age–sex patient registers, and allowed regular searches of their computerized diagnostic coding systems. All practices refer patients to a single secondary care hospital.

Case ascertainment was by prospective daily searches for acute events in hospital (hot pursuit) and retrospective searches of hospital, primary care administrative and diagnostic coding data, and centralized death certification (cold pursuit) for cases missed by hot pursuit and deaths in the community. Full details of case ascertainment are available in Appendix S1 (supporting information). Following ascertainment, a study clinician assessed patients as soon as possible after the event. Informed consent was sought, where possible, or obtained from a relative. A standard clinical history and examination was recorded, along with details of medication, past medical history, all investigations relevant to the admission and all interventions occurring subsequent to the event. All diagnoses were reviewed subsequently by a senior clinician. After this, aortic events with any ongoing diagnostic uncertainty were assessed by a separate consultant vascular surgeon to ensure agreement on classification. If a patient died before assessment, or was identified only by cold pursuit, eye‐witness accounts were obtained and relevant records reviewed. If death occurred outside hospital, or before investigation, autopsy results were also reviewed. Clinical details of all deaths with possible vascular aetiology were sought from primary care physicians or other clinicians.

All surviving patients were followed up by a research nurse at 6 months or via their family doctor, with recurrent events also identified from ongoing study surveillance. If a recurrent vascular event was suspected, the patient was assessed by a study physician. Premorbid disability and disability on follow‐up were assessed with the modified Rankin scale^42^. All study clinicians were trained in performing modified Rankin scale assessments by completing the approved digital training course.

To assess the potential impact of AAA screening in relation to risk factors documented in primary care, data from primary healthcare records for all cases and for all of the underlying study population were obtained (individual patient data for cases and age–sex‐specific tabular data for the population) on history of hypertension and smoking status. The accuracy of these data was tested by questioning of patients and relatives, and review of records. In addition, all premorbid BP measurements were obtained from primary care records of incident cases (available for 98·1 per cent). These were taken using automated sphygmomanometers with the patient seated. A cut‐off value of 140/90 mmHg was used to define hypertension.

### Analysis

All patients with acute vascular events affecting the aorta from 1 April 2002 to 31 March 2014 were assessed. Patients who had an event while temporarily away from Oxfordshire were included, but visitors to Oxfordshire who were not registered with one of the study practices were excluded. AAA events were classified as either ruptured AAA or symptomatic AAA, and defined by involvement of the aorta at or below the level of the renal arteries. Symptomatic AAAs were defined as acutely symptomatic, resulting in the need for emergency medical attention. These normally presented with acute severe pain in the abdomen, back or flank not obviously attributable to another cause and without evidence of AAA rupture on imaging^43^. No strict aortic diameter was required for inclusion, but all patients had an aortic diameter greater than 5·0 cm. Aortic diameter was recorded from either the most recent or emergency department CT or ultrasound image, at the time of emergency repair by the vascular surgeon if the patient went straight to the operating theatre, or at autopsy.

All AAA events were categorized as first‐ever incident or recurrence. A rupture in a patient with a previous symptomatic AAA was classified as a recurrent event if the delay before rupture was more than 48 h. The population structure was derived from the general practice age–sex registers and based on the mean of the ten mid‐year population age–sex structures. Age‐ and sex‐specific rates (per 100 000 population per year) were calculated for all events of each type, and for incident events. Numerical data were expressed as mean(s.d.) or proportions, as appropriate. Group differences in continuous variables were examined with Student's t test or Mann–Whitney U test for parametric and non‐parametric variables respectively. Group differences in categorical variables were examined with Fisher's exact test or χ^2^ test, as appropriate. Survival rates were derived by Kaplan–Meier analysis. Analyses were done with SPSS® version 20.0 (IBM, Armonk, New York, USA). P < 0·050 was considered statistically significant.

## Results

Of the 92 728 study population, 23 808 were aged 55 years or above, of whom 8954 (37·6 per cent) had diagnosed hypertension and 11 480 (48·2 per cent) were ever‐smokers. A total of 196 acute non‐occlusive aortic events occurred in 179 patients, including 127 acute aneurysms (108 abdominal, 6 thoracoabdominal, 13 thoracic) and 69 acute dissections (51 Stanford type A, 18 type B). A total of 103 incident acute AAA events were analysed (rate 9 per 100 000 per year; 72·8 per cent men; mean age 78·7 years). Seventy‐nine (76·7 per cent) were incident ruptured AAAs (7 per 100 000 per year) and 24 (23·3 per cent) were acutely symptomatic AAAs (2 per 100 000 per year); 61 were fatal (5 per 100 000 per year). Of the 103 incident acute AAA events, 31 (30·1 per cent) caused sudden death in the community and a further six patients (5·8 per cent) died in transit to hospital or shortly after arrival. Of the remaining 66 patients, 43 (65 per cent) had emergency surgery (37 open and 6 endovascular repair).

**Figure 1:**
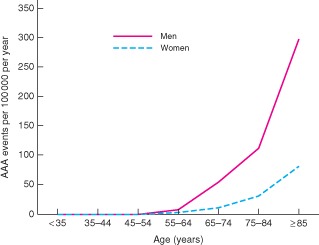
Age‐specific rates per 100 000 population per year for incident acute abdominal aortic aneurysm (AAA) events in men and women, 2002–2014

The annual incidence of incident acute AAA events was 55 per 100 000 in men aged 65–74 years, but higher in older men (75–85 years: 112 per 100 000; 85 years or above: 298 per 100 000) and older women (age 85 years or above: 82 per 100 000) (Fig. 1). Only 22·3 per cent of incident events occurred in men aged 65–74 years (Table 1), and 13 per cent (8 of 61) of aneurysm‐related deaths. Some 33 of the 35 events at age below 75 years occurred in current (24) or previous (9) smokers (Table 1), with a higher prevalence of current smokers (P < 0·001) in patients with acute events than in the underlying study population (Fig. 3 and Table S1, supporting information). The prevalence of current or previous smoking was lower in women with an acute AAA event (ever‐smokers: 61 per cent in women versus 83 per cent in men; P = 0·019) (Table S2, supporting information). In addition, the mean age at event was lowest in current smokers, compared with ex‐smokers and never‐smokers (P < 0·001) (Table S3, supporting information). For those who had given up smoking at least 10 years previously, age at event was similar to that for never‐smokers. AAA acute event rates were greatest (274 per 100 000 per year) in male current smokers aged 65–74 years (Table 1), with low rates found for non‐smokers in this age group (6 per 100 000 per year).

**Table 1:** Age‐ and sex‐specific rates per 100 000 population per year for incident acute abdominal aortic aneurysm events in the total population, current smokers, ex‐smokers and patients with hypertension, 2002–2014

	Age (years)					Total
	45–54	55–64	65–74	75–84	≥ 85	
Total population						
Men	0	5	23	28	19	75
Rate per 100 000 per year	–	8 (3, 19)	55 (35, 82)	112 (75, 162)	298 (179, 465)	13 (10, 17)
Women	0	2	5	10	11	28
Rate per 100 000 per year	–	3 (0, 12)	11 (4, 26)	31 (15, 58)	82 (41, 146)	5 (3, 7)
Total	0	7	28	38	30	103
Rate per 100 000 per year	–	6 (2, 12)	32 (22, 47)	67 (47, 92)	151 (102, 216)	9 (8, 11)
Current smokers						
Men	0	4	17	6	2	29
Rate per 100 000 per year	–	34 (9, 88)	274 (159, 438)	151 (56, 329)	209 (25, 755)	27 (18, 39)
Women	0	1	2	3	0	6
Rate per 100 000 per year	–	12 (0, 64)	45 (5, 163)	66 (14, 191)	–	7 (3, 15)
Total	0	5	19	9	2	35
Rate per 100 000 per year	–	25 (8, 58)	179 (108, 279)	105 (48, 200)	72 (9, 261)	18 (12, 25)
Ex‐smokers						
Men	0	1	5	14	13	33
Rate per 100 000 per year	–	6 (0, 36)	29 (9, 67)	124 (68, 208)	452 (240, 772)	34 (24, 48)
Women	0	0	3	4	4	11
Rate per 100 000 per year	–	–	20 (4, 58)	31 (9, 80)	84 (23, 214)	12 (6, 21)
Total	0	1	8	18	17	44
Rate per 100 000 per year	–	3 (0, 18)	25 (11, 48)	75 (44, 118)	222 (129, 355)	23 (17, 31)
Patients with diagnosed hypertension						
Men	0	2	13	15	14	44
Rate per 100 000 per year	–	13 (2, 48)	72 (38, 124)	119 (66, 196)	360 (197, 604)	71 (51, 95)
Women	0	2	4	10	10	26
Rate per 100 000 per year	–	15 (2, 56)	21 (6, 55)	57 (27, 104)	128 (61, 235)	37 (24, 55)
Total	0	4	17	25	24	70
Rate per 100 000 per year	–	14 (4, 37)	46 (27, 74)	82 (53, 122)	205 (131, 305)	53 (41, 67)

Values in parentheses are 95 per cent c.i.

**Figure 2:**
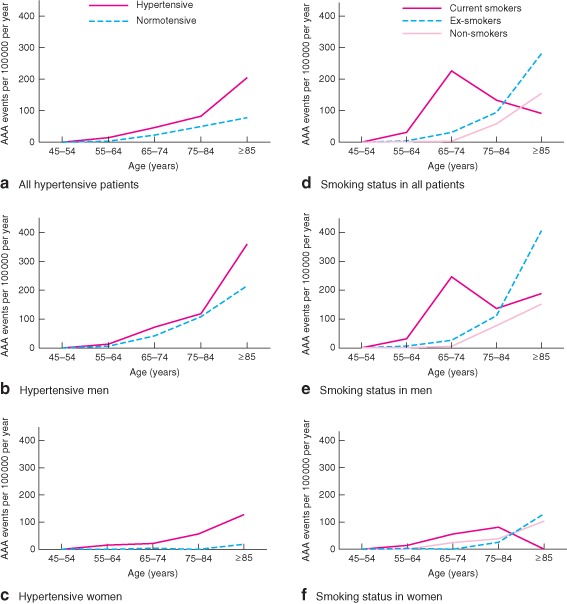
Age‐specific rates per 100 000 population per year for incident acute abdominal aortic aneurysm (AAA) events in **a,d** all patients, **b,e** men and **c,f** women according to **a–c** hypertensive and **d–f** smoking status, 2002–2014. The term ‘normotensive’ refers to no diagnosis of hypertension before the AAA event

**Figure 3:**
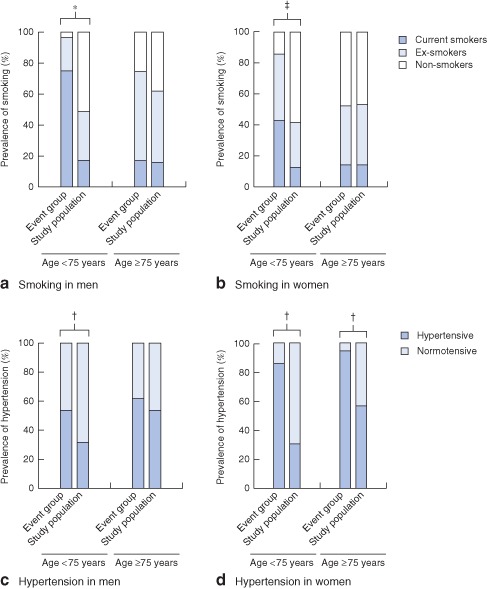
Age‐ and sex‐specific prevalence of **a,b** smoking and **c,d** hypertension in the incident abdominal aortic aneurysm event group and the total Oxford Vascular (OXVASC) Study population, 2002–2014. *P < 0·001, †P = 0·010, ‡P = 0·050 (χ^2^ test)

In contrast to current smoking, which was a powerful risk factor for premature AAA events in both men and women (*Figs* 2 and 3), hypertension was the predominant risk factor in women at all ages. Only two (7 per cent) of 28 women with an acute AAA were normotensive, compared with 31 (41 per cent) of 75 men (*P* < 0·001). Consequently, among women with hypertension the incidence of acute AAA reached 57 per 100 000 per year at age 75–84 years and 128 per 100 000 per year at age 85 years and above (*Table* 1 and *Fig*. 2). Premorbid control of BP was also worse in women. Over the 5 years before the incident event, the mean(s.d.) proportion of all pressures recorded in primary care (mean of 12 readings per patient) that were greater than 140/90 mmHg was 53·0(27·0) per cent in women and 36·1(31·7) per cent in men (*P* = 0·016).

The acute AAA incidence rates in the present study were compared with those derived from Hospital Episode Statistics (HES) data and death certification over the same interval for both the local Oxfordshire population and the UK. Some 41·7 per cent of incident acute AAA events were missed by routine HES and mortality coding when using the acute AAA code (I713), mainly owing to incorrect coding as elective procedures, unspecified aneurysm or thoracic aneurysm hospital episodes (*n* = 35), or as acute coronary syndromes or aortic dissection events (*n* = 8) (*Fig. S1*, supporting information). Of patients who reached hospital, only 34 of 66 were coded correctly. Coding was least accurate for acutely symptomatic aneurysms: 25 per cent were coded correctly compared with 67 per cent of ruptured AAAs (*P* < 0·001). Use of all acute aneurysm codes (I711, I713, I715, I718) increased the number of correctly identified cases to 67·0 per cent (78 per cent for aneurysm‐related deaths), but at the expense of an increased rate of false positives (16 per cent), due mainly to the inclusion of non‐abdominal aneurysms. No significant improvement in coding analysis was detected throughout the study.

The overall 30‐day case fatality rate was 59·2 per cent for incident acute AAAs and 42 per cent for patients who reached hospital alive. The 30‐day fatality rate was higher for patients with a ruptured AAA than in those with an acutely symptomatic AAA (75 *versus* 8 per cent respectively; *P* < 0·001). The 30‐day case fatality rate due to acute AAA increased from 40 per cent at age less than 75 years to 69 per cent at age 75 years or above (*P* = 0·012) (*Table S4*, supporting information). For all patients, the mean(s.d.) duration of stay was 9·3(15·5) days and intensive care unit (ICU) stay was 4·8(10·7) days; respective stays were 15·3(17·5) and 7·9(12·9) days for those who reached hospital alive. For 40 patients who were discharged from hospital alive, the respective durations were 19·3(15·8) and 6·9(7·3) days, and were similar for patients aged 75 years or more and those aged less than 75 years (16·4(13·5) *versus* 21·8(17·6) days respectively; *P* = 0·360). Five patients required community hospital rehabilitation and two needed permanent care home placement; all of these patients were aged 75 years or above.

Of the 103 patients with acute AAA, 15 (14·6 per cent) had a known AAA under surveillance. The mean(s.d.) age of these patients at the AAA event was 81·3(8·3) years, and 77·0(9·8) years at diagnosis. Ten presented with a ruptured AAA and five with an acutely symptomatic aneurysm. Five died before reaching hospital, and eight died within 30 days of the event.

## Discussion

This prospective population‐based study examined the incidence, risk factors and outcome of all acute AAA events, without exclusion of patients by age or sex. This enabled assessment of the impact of factors on the risk of an acute AAA event (symptomatic or ruptured), as opposed to the prevalence of asymptomatic AAA. There are several important findings. First, the incidences of acute AAA (9 per 100 000 per year) and related death rates (5 per 100 000 per year) were 30–40 per cent higher than local and national estimates based on routine coding data^16^, ^17^, ^18^. Second, despite the above, the incidence of ruptured AAA in men aged 65–74 years was lower than that found in the MASS trial (55 per 100 000 per year here *versus* 96 pre 100 000 per year in the MASS control group; *P* = 0·024), in keeping with recent data suggesting that AAA admission and death rates have fallen in this age group over the past two decades^16^, ^17^. Third, nearly two‐thirds of all acute AAA events now occur at age 75 years or above, when the events have a higher risk of death. These age‐ and risk factor‐specific incidence and outcome rates provide valuable data for informing future AAA screening strategy.

This study suggests that current screening programmes should be extended to men at older ages. There was a much higher incidence of acute AAA events in men aged 75–84 years than in those aged 64–75 years in Oxfordshire. The increase in 30‐day case fatality of acute AAA events with age was significant. Male smokers aged 65 years are the principal target of the current screening programme, as incidence rates of AAA rupture in non‐smokers aged 65–74 years are low. Indeed, the mean age of acute AAA in non‐smokers was nearly 20 years more, by which time any benefit from screening may be lost. Overall, approximately one‐third of all acute AAA events occurred at age 85 years or above. Although screening this elderly population could be controversial, and life expectancy after surgery at this age is much shorter, longer‐term survival might increase in future and screening of selected individuals might be appropriate. In terms of targeting screening of the elderly, hypertension continued to be a risk factor for acute AAA but smoking was less strongly associated.

Current smoking was particularly strongly associated with the occurrence of an acute AAA at younger ages, whereas hypertension was an important risk factor in women, being present in 93 per cent (26 of 28) of those with incident acute AAA. More than one‐quarter of acute AAA events at all ages occurred in women, and so consideration should be given to offering screening to women with risk factors; a policy of offering screening to all women aged 75 years with known hypertension might be evaluated in a randomized trial.

The strong influence of smoking and hypertension on risk of AAA rupture, and their apparent interactions with age and sex, are consistent with previous studies of rupture during follow‐up of patients with AAA^27^, ^28^, ^29^, ^35^. Although current smoking increases the rate of expansion of AAA by 15–20 per cent, it doubles the risk of AAA rupture^29^. The present study reveals that men aged 65–74 years who smoke have about a 3 per cent 10‐year risk of acute AAA, highlighting the need for screening campaigns to reach this high‐risk group. There was a much lower risk in ex‐smokers, suggesting that smoking cessation should be strongly encouraged in men with a small AAA or those unfit for surgery. Hypertension also appeared to have little effect on the rate of AAA expansion in previous cohort studies, but increased the risk of rupture by 30 per cent per 10‐mmHg increase in mean BP^29^. The strong association between hypertension and risk of acute AAA in women in the present study might explain why, although women have a lower prevalence of AAA, they have a fourfold increase in the risk of subsequent rupture, adjusted for aneurysm diameter^29^. Not only were almost all women with an acute AAA in the present study hypertensive, but premorbid control of BP was also substantially worse than in men, highlighting the importance of active management of hypertension in women with a small AAA.

There are a number of potential shortcomings in the present study. Although the OXVASC population covers a broad range of deprivation, with 22 per cent of districts ranking in the lower third nationally, the population is 94 per cent white Caucasian and the proportion of other racial groups is small (3·1 per cent Asian, 1·5 per cent Chinese and 1·4 per cent Afro‐Caribbean)^44^. The proportion of white caucasians is similar to that in the UK as a whole (88 per cent) and in many other Western countries (Australia 90 per cent, France 91 per cent, Germany 93·9 per cent). Although demographic differences between Oxfordshire and the rest of the UK might undermine the generalizability of the present findings, the coding‐based incidence of acute AAA in OXVASC was similar to that for the UK as a whole (*Fig. S1*, supporting information). Despite overlapping methods it is unlikely that every single acute aortic event in the Oxfordshire population was ascertained. Some sudden deaths from acute AAA in the community undoubtedly go undiagnosed. However, such underascertainment is likely to be greater at older ages where post‐mortem examination is less routine; the estimate of the proportion of AAA‐related deaths at older ages may be conservative. Finally, the main analyses in the present study were based on only 103 acute AAA events, inevitably limiting the precision of some of the estimates of rates and risk factor associations. National‐level studies of routine coding data have the advantage of larger numbers, but are likely to miss over 40 per cent of incident events, particularly acutely symptomatic AAAs.

**Supporting information**

Additional supporting information may be found in the online version of this article:

**Appendix S1** Case ascertainment: hot and cold pursuit methodology (Word document)

**Fig. S1** Efficacy of routine local and national coding (Hospital Episode Statistics data and death certification) in identifying acute abdominal aortic aneurysm events compared with Oxford Vascular (OXVASC) Study ascertainment (Word document)

**Table S1** Comparison of the presence of risk factors in patients with acute events *versus* the background study population expressed as age‐ and sex‐adjusted risk ratios (Word document)

**Table S2** Demographics and risk factors for incident acute abdominal aortic aneurysm by sex and type (Word document)

**Table S3** Smoking status and age of patient at acute event, stratified by hypertensive status (Word document)

**Table S4** Events and aneurysm‐related mortality rates by age and risk factor status in the Oxford Vascular (OXVASC) Study, and current UK figures for aneurysm‐related mortality following elective aneurysm repair (Word document)

## Supporting information


**Appendix S1** Case ascertainment: hot and cold pursuit methodology

Efficacy of routine local and national coding (Hospital Episode Statistics (HES) data and death certification) in identifying acute abdominal aortic aneurysm (AAA) events compared with Oxford Vascular (OXVASC) Study ascertainment. OXVASC ascertainment is taken as the standard. I713 is the ICD‐10 code for acute/ruptured AAA; I711, I713, I715 and I718 codes refer to acute/ruptured aortic aneurysms at other anatomical locations. Only incident events were analysed. For specificity calculations, the total number of OXVASC incident acute aortic events (179) during the 12‐year study interval was used

Comparison of the presence of risk factors in patients with acute events versus the background study population expressed as age‐ and sex‐adjusted risk ratios

Demographics and risk factors for incident acute abdominal aortic aneurysm by sex and type

Smoking status and age of patient at acute event, stratified by hypertensive status

Events and aneurysm‐related mortality rates by age and risk factor status in the Oxford Vascular (OXVASC) Study, and current UK figures for aneurysm‐related mortality following elective aneurysm repair
