# Supplementary material for: Age‐specific incidence, risk factors and outcome of acute abdominal aortic aneurysms in a defined population
Source: Br J Surg. 2015 May 7;102(8):907–15. doi: 10.1002/bjs.9838 (PMC4687424; doi:10.1002/bjs.9838)
Supplement: Supplementary file 1 — Appendix S1 Case ascertainment: hot and cold pursuit methodology [file bjs0102-0907-sd4.doc]

**Appendix S1** Case ascertainment: hot and cold pursuit methodology

Hot pursuit was based on the daily assessment of all patients with a possible vascular event identified by: (1) daily searches of emergency department admission and symptom/diagnosis registers; (2) daily listing from the central admissions department of all patients from our general practices admitted to hospital, and assessment of these patients in hospital; (3) daily visits to the cardiac surgery and vascular surgery wards and review of daily lists of all patients referred to vascular surgery; (4) daily identification via bereavement officers of patients dead on arrival at hospital or who died soon after; (5) daily assessment of all patients undergoing diagnostic angiographic, angioplasty/stenting or arterial surgical procedures in any territory.

The methods of cold pursuit were: (1) weekly review of all listed surgical procedures undertaken by vascular and cardiovascular surgery; (2) direct collection of general practice records and diagnoses from each individual practice on a monthly basis – all relevant vascular diagnoses made in primary care are assessed by a senior clinician within the study and the patient event ascertained; (3) monthly practice-specific list of all patients with relevant diagnostic codes from the coding departments covering all acute and community hospitals (Hospital Episode Statistics (HES) data); (4) monthly visits to the coroner’s office to review out-of-hospital deaths; (5) review of all death certificates and relevant clinical details in the study practices; (6) practice-specific listings of all ICD-10 death codes from the local Department of Public Health; (7) review of vascular surgery outpatient clinic letters to identify patients who were not admitted to hospital. For all cases not initially identified by HES data and death certification, these data sources were re-searched using National Health Service number and other identifiers, where possible.
