# Supplementary material for: Age‐specific incidence, risk factors and outcome of acute abdominal aortic aneurysms in a defined population
Source: Br J Surg. 2015 May 7;102(8):907–15. doi: 10.1002/bjs.9838 (PMC4687424; doi:10.1002/bjs.9838)
Supplement: Supplementary file 2 — Efficacy of routine local and national coding (Hospital Episode Statistics (HES) data and death certification) in identifying acute abdominal aortic aneurysm (AAA) events compared with Oxford Vascular (OXVASC) Study ascertainment. OXVASC ascertainment is taken as the standard. I713 is the ICD‐10 code for acute/ruptured AAA; I711, I713, I715 and I718 codes refer to acute/ruptured aortic aneurysms at other anatomical locations. Only incident events were analysed. For specificity calculations, the total number of OXVASC incident acute aortic events (179) during the 12‐year study interval was used [file bjs0102-0907-sd6.doc]

Incidence per 100 000 population


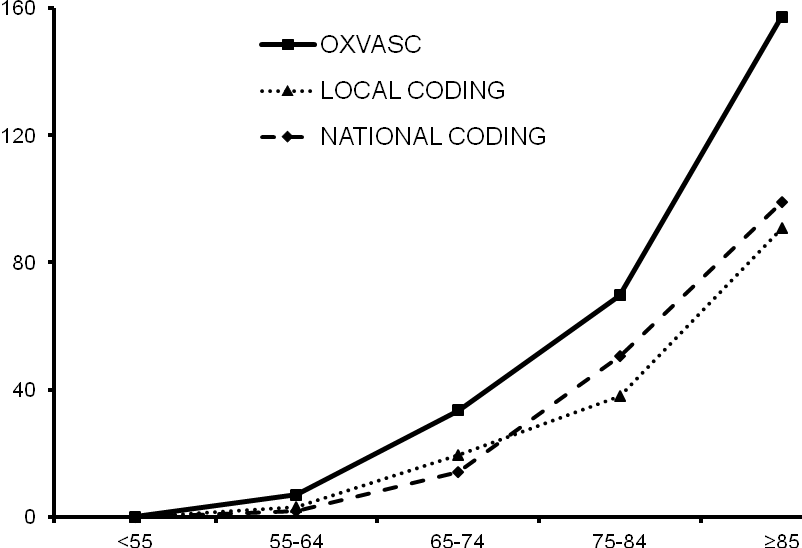


Age (years)

Sensitivity and specificity of local routine coding compared with OXVASC ascertainment

|  | ICD-10 | | OXVASC ascertainment |
| --- | --- | --- | --- |
|  | I713  (any mention) | I711, I713, I715, I718  (any mention) |  |
| Total incident events identified | 61 | 82 | 103 |
| Correctly identified incident events | 60 (98) | 69 (84) | 103 (100) |
| Incorrectly identified incident events | 1 (2) | 13 (16) | 0 (0) |
| Sensitivity | 60 of 103 (58.3) | 69 of 103 (67.0) | 103 (100) |
| Specificity | 75 of 76 (98.7) | 63 of 76 (82.9) | 76 of 76 (100) |
| Positive predictive value (%) | 97.4 | 77.1 | 100 |
| Negative predictive value (%) | 69.2 | 70.4 | 100 |

Values in parentheses are percentages.

**Fig. S1** Efficacy of routine local and national coding (Hospital Episode Statistics (HES) data and death certification) in identifying acute abdominal aortic aneurysm (AAA) events compared with Oxford Vascular (OXVASC) Study ascertainment. OXVASC ascertainment is taken as the standard. I713 is the ICD-10 code for acute/ruptured AAA; I711, I713, I715 and I718 codes refer to acute/ruptured aortic aneurysms at other anatomical locations. Only incident events were analysed. For specificity calculations, the total number of OXVASC incident acute aortic events (179) during the 12-year study interval was used
