# Supplementary material for: Age‐specific incidence, risk factors and outcome of acute abdominal aortic aneurysms in a defined population
Source: Br J Surg. 2015 May 7;102(8):907–15. doi: 10.1002/bjs.9838 (PMC4687424; doi:10.1002/bjs.9838)
Supplement: Supplementary file 3 — Comparison of the presence of risk factors in patients with acute events versus the background study population expressed as age‐ and sex‐adjusted risk ratios [file bjs0102-0907-sd3.doc]

**Table S1** Comparison of the presence of risk factors in patients with acute events *versus* the background study population expressed as age- and sex-adjusted risk ratios

|  | No. with risk factor in event group | No. of risk factor in study population | Risk ratio | *P** |
| --- | --- | --- | --- | --- |
| Aged 55–74 years |  |  |  |  |
| Men |  |  |  |  |
| Ever smoker | 27 of 28 | 4248 of 8717 | 1.98 (1.84, 2.13) | < 0.001 |
| Current smoker | 21 of 28 | 1492 of 8717 | 4.38 (3.52, 5.45) | < 0.001 |
| Hypertension | 15 of 28 | 2755 of 8717 | 1.70 (1.20, 2.40) | 0.003 |
| Women |  |  |  |  |
| Ever smoker | 6 of 7 | 3604 of 8699 | 2.07 (1.53, 2.80) | < 0.001 |
| Current smoker | 3 of 7 | 1099 of 8699 | 3.39 (1.44, 7.99) | 0.005 |
| Hypertension | 6 of 7 | 2656 of 8699 | 2.81 (2.07, 3.81) | < 0.001 |
| Aged ≥ 75 years |  |  |  |  |
| Men |  |  |  |  |
| Ever smoker | 35 of 47 | 1616 of 2609 | 1.20 (1.01, 1.43) | 0.029 |
| Current smoker | 8 of 47 | 416 of 2609 | 1.07 (0.56, 2.02) | 0.842 |
| Hypertension | 29 of 47 | 1399 of 2609 | 1.15 (0.92, 1.45) | 0.231 |
| Women |  |  |  |  |
| Ever smoker | 11 of 21 | 2012 of 3783 | 0.98 (0.65, 1.48) | 0.936 |
| Current smoker | 3 of 21 | 537 of 3783 | 1.01 (0.35, 2.88) | 0.993 |
| Hypertension | 20 of 21 | 2144 of 3783 | 1.68 (1.52, 1.86) | < 0.001 |
| All aged ≥ 55 years |  |  |  |  |
| Men |  |  |  |  |
| Ever smoker | 62 of 75 | 5864 of 11 326 | 1.60 (1.44, 1.77) | < 0.001 |
| Current smoker | 29 of 75 | 1908 of 11 326 | 2.30 (1.72, 3.06) | < 0.001 |
| Hypertension | 44 of 75 | 4154 of 11 326 | 1.60 (1.32, 1.94) | < 0.001 |
| Women |  |  |  |  |
| Ever smoker | 17 of 28 | 5616 of 12 482 | 1.35 (1.00, 1.82) | 0.045 |
| Current smoker | 6 of 28 | 1636 of 12 482 | 1.63 (0.80, 3.33) | 0.180 |
| Hypertension | 26 of 28 | 4800 of 12 482 | 2.41 (2.17, 2.68) | <0.001 |

Values in parentheses are 95 per cent c.i. *χ^2^ test.
