# Supplementary material for: Age‐specific incidence, risk factors and outcome of acute abdominal aortic aneurysms in a defined population
Source: Br J Surg. 2015 May 7;102(8):907–15. doi: 10.1002/bjs.9838 (PMC4687424; doi:10.1002/bjs.9838)
Supplement: Supplementary file 4 — Demographics and risk factors for incident acute abdominal aortic aneurysm by sex and type [file bjs0102-0907-sd5.doc]

**Table S2** Demographics and risk factors for incident acute abdominal aortic aneurysm by sex and type

|  | Total (*n* = 103) | Men (*n* = 75) | Women (*n* = 28) | *P** | Ruptured (*n* = 79) | Symptomatic (*n* = 24) | *P** |
| --- | --- | --- | --- | --- | --- | --- | --- |
| Mean(s.d.) age (years) | 78.7(8.6) | 78.0(8.5) | 80.6(8.8) | 0.168† | 78.8(9.2) | 78.2(6.5) | 0.764† |
| Men | 75 (72.8) |  |  |  | 59 (75) | 16 (67) | 0.439 |
| Previous vascular disease |  |  |  |  |  |  |  |
| Angina | 32 (31.1) | 23 (31) | 9 (32) | 0.885 | 25 (32) | 7 (29) | 0.818 |
| Acute coronary syndrome | 25 (24.3) | 21 (28) | 4 (14) | 0.199 | 19 (24) | 6 (25) | 0.924 |
| Transient ischaemic attack | 18 (17.5) | 14 (19) | 4 (14) | 0.773 | 14 (18) | 4 (17) | 0.905 |
| Stroke | 18 (17.5) | 14 (19) | 4 (14) | 0.773 | 12 (15) | 6 (25) | 0.268 |
| Peripheral arterial disease | 19 (18.4) | 12 (16) | 7 (25) | 0.295 | 12 (15) | 7 (29) | 0.122 |
| Any | 59 (57.3) | 41 (55) | 18 (64) | 0.380 | 45 (57) | 14 (58) | 0.905 |
| Risk factors |  |  |  |  |  |  |  |
| Current smoker | 35 (34.0) | 29 (39) | 6 (21) | 0.100 | 25 (32) | 10 (42) | 0.36 |
| Ever smoker | 79 (76.7) | 62 (83) | 17 (61) | 0.019 | 60 (76) | 19 (79) | 0.744 |
| Hypertension | 70 (68.0) | 44 (59) | 26 (93) | 0.001 | 51 (65) | 19 (79) | 0.179 |
| Diabetes mellitus | 11 (10.7) | 8 (11) | 3 (11) | 1.000 | 8 (10) | 3 (13) | 0.715 |
| Cardiac failure | 14 (13.6) | 8 (11) | 6 (21) | 0.156 | 12 (15) | 2 (8) | 0.512 |
| Atrial fibrillation | 20 (19.4) | 15 (20) | 5 (18) | 0.807 | 17 (22) | 3 (13) | 0.393 |
| Medications |  |  |  |  |  |  |  |
| Statin | 45 (43.7) | 32 (43) | 13 (46) | 0.731 | 33 (42) | 12 (50) | 0.477 |
| Aspirin | 44 (42.7) | 33 (44) | 11 (39) | 0.667 | 33 (42) | 11 (46) | 0.725 |
| Other antiplatelet agent | 4 (3.9) | 3 (4) | 1 (4) | 1.000 | 2 (3) | 2 (8) | 0.231 |
| Warfarin | 10 (9.7) | 8 (11) | 2 (7) | 0.724 | 9 (11) | 1 (4) | 0.446 |
| Antihypertensive drug |  |  |  | 0.004 |  |  | 0.429 |
| 0 | 31 (30.1) | 29 (39) | 2 (7) |  | 26 (33) | 5 (21) |  |
| 1 | 24 (23.3) | 14 (19) | 10 (36) |  | 18 (23) | 6 (25) |  |
| ≥ 2 | 48 (46.6) | 32 (43) | 16 (57) |  | 35 (44) | 13 (54) |  |

Values in parentheses are percentages unless indicated otherwise. *Fisher’s exact test or χ^2^ test, except †Student’s *t* test.
