# Supplementary material for: Age‐specific incidence, risk factors and outcome of acute abdominal aortic aneurysms in a defined population
Source: Br J Surg. 2015 May 7;102(8):907–15. doi: 10.1002/bjs.9838 (PMC4687424; doi:10.1002/bjs.9838)
Supplement: Supplementary file 5 — Smoking status and age of patient at acute event, stratified by hypertensive status [file bjs0102-0907-sd2.doc]

**Table S3** Smoking status and age of patient at acute event, stratified by hypertensive status

Values in parentheses are percentages unless indicated otherwise; *values are mean(s.d.). †ANOVA, except ‡χ^2^ test.

|  | Lifelong non-smoker | Ex-smoker | | | Current smoker | *P*† | Total |
| --- | --- | --- | --- | --- | --- | --- | --- |
|  |  | For > 10 years | For < 10 years | All |  |  |  |
| No. of patients | 24 (23.3) | 29 (28.2) | 15 (14.6) | 44 (42.8) | 35 (34.0) |  | 103 |
| Men | 13 (54) | 24 (83) | 9 (60) | 33 (75) | 29 (83) | 0.028‡ | 75 (72.8) |
| No. with hypertension | 16 (66.7) | 21 (72.4) | 11 (73.3) | 32 (72.7) | 22 (62.9) | 0.831‡ | 70 (68.0) |
| Age (years)* | 83.3(7.9) | 83.7(6.7) | 76.9(5.1) | 81.2(7.0) | 72.2(7.2) | < 0.001 | 78.7(8.6) |
| Patients with hypertension | 84.4(8.8) | 83.7(5.6) | 77.1(5.8) | 81.2(6.5) | 72.9(7.9) | < 0.001 | 79.4(8.7) |
| Patients without hypertension | 81.2(5.8) | 83.8(9.4) | 76.3(3.1) | 81.3(8.5) | 70.8(5.7) | 0.001 | 77.1(8.4) |
