# Supplementary material for: Age‐specific incidence, risk factors and outcome of acute abdominal aortic aneurysms in a defined population
Source: Br J Surg. 2015 May 7;102(8):907–15. doi: 10.1002/bjs.9838 (PMC4687424; doi:10.1002/bjs.9838)
Supplement: Supplementary file 6 — Events and aneurysm‐related mortality rates by age and risk factor status in the Oxford Vascular (OXVASC) Study, and current UK figures for aneurysm‐related mortality following elective aneurysm repair [file bjs0102-0907-sd1.doc]

**Table S4** Events and aneurysm-related mortality rates by age and risk factor status in the Oxford Vascular (OXVASC) Study, and current UK figures for aneurysm-related mortality following elective aneurysm repair

|  | < 75 years | 75–84 years | ≥ 85 years | *P* | Total |
| --- | --- | --- | --- | --- | --- |
| Events | 35 | 38 | 30 |  | 103 |
| Out-of-hospital death | 6 (17) | 15 (39) | 10 (33) | 0.101 | 31 (30.1) |
| Aneurysm-related death | 14 (40) | 25 (66) | 22 (73) | 0.012 | 61 (59.2) |
| Men | 28 (80) | 28 (74) | 19 (63) | 0.141 | 75 (72.8) |
| Aneurysm-related death | 10 (36) | 19 (68) | 16 (84) | 0.002 | 45 (60) |
| Women | 7 (20) | 10 (26) | 11 (37) | 0.137 | 28 (27.2) |
| Aneurysm-related death | 4 (57) | 6 (60) | 6 (55) | 0.970 | 16 (57) |
| Ever-smokers | 33 (94) | 27 (71) | 19 (63) | 0.008 | 79 (76.7) |
| Aneurysm-related death | 13 (39) | 17 (63) | 15 (79) | 0.016 | 45 (57) |
| Current smokers | 24 (69) | 9 (24) | 2 (7) | < 0.001 | 35 (34.0) |
| Aneurysm-related death | 8 (33) | 7 (78) | 1 (50) | 0.071 | 16 (46) |
| Non-smokers | 2 (6) | 11 (29) | 11 (37) | 0.008 | 24 (23.3) |
| Aneurysm-related death | 1 (50) | 8 (73) | 7 (64) | 0.792 | 16 (67) |
| Hypertensive | 21 (60) | 25 (66) | 24 (80) | 0.208 | 70 (68.0) |
| Aneurysm-related death | 9 (43) | 17 (68) | 17 (71) | 0.107 | 43 (61) |
| Normotensive | 14 (40) | 13 (34) | 6 (20) | 0.214 | 33 (32.0) |
| Aneurysm-related death | 5 (36) | 8 (62) | 5 (83) | 0.115 | 18 (55) |
| Current UK figures for aneurysm-related mortality following elective AAA repair* | | | |  |  |
| Aneurysm-related death (%) | 2 | 4 | 6 |  |  |

Values in parentheses are percentages.

***References**

Grant SW, Hickey GL, Grayson AD, Mitchell DC, McCollum CN. National risk prediction model for elective abdominal aortic aneurysm repair. *Br J Surg* 2013; **100**: 645–653.

Raval MV, Eskandari MK. Outcomes of elective abdominal aortic aneurysm repair among the elderly: endovascular *versus* open repair. *Surgery* 2012; **151**: 245–260.

Choke E, Lee K, McCarthy M, Nasim A, Naylor AR, Bown M, Sayers R. Risk models for mortality following elective open and endovascular abdominal aortic aneurysm repair: a single institution experience. *Eur J Vasc Endovasc Surg* 2012; **44**: 549–554.

Saratzis A, Mohamed S. Endovascular abdominal aortic aneurysm repair in the geriatric population. *J Geriatr Cardiol* 2012; **9**: 285–291.
